# TRANS-CNN-Based Gesture Recognition for mmWave Radar

**DOI:** 10.3390/s24061800

**Published:** 2024-03-11

**Authors:** Huafeng Zhang, Kang Liu, Yuanhui Zhang, Jihong Lin

**Affiliations:** College of Mechanical and Electrical Engineering, China Jiliang University, Hangzhou 310018, China; zhanghf@cjlu.edu.cn (H.Z.); zyh@cjlu.edu.cn (Y.Z.); jhlin@cjlu.edu.cn (J.L.)

**Keywords:** mmWave radar, dynamic gesture recognition, multi-head self-attention mechanism, TRANS-CNN, point cloud

## Abstract

In order to improve the real-time performance of gesture recognition by a micro-Doppler map of mmWave radar, the point cloud based gesture recognition for mmWave radar is proposed in this paper. Two steps are carried out for mmWave radar-based gesture recognition. The first step is to estimate the point cloud of the gestures by 3D-FFT and the peak grouping. The second step is to train the TRANS-CNN model by combining the multi-head self-attention and the 1D-convolutional network so as to extract the features in the point cloud data at a deeper level to categorize the gestures. In the experiments, TI mmWave radar sensor IWR1642 is used as a benchmark to evaluate the feasibility of the proposed approach. The results show that the accuracy of the gesture recognition reaches 98.5%. In order to prove the effectiveness of our approach, a simply 2Tx2Rx radar sensor is developed in our lab, and the accuracy of recognition reaches 97.1%. The results show that our proposed gesture recognition approach achieves the best performance in real time with limited training data in comparison with the existing methods.

## 1. Introduction

In the field of intelligent interaction [1], gesture recognition is used extensively in applications such as smart homes [2,3], robot control [4], autonomous driving [5], and AR/VR [6]. At present, gesture recognition technologies by wearable devices [7] or vision sensors [8] are well developed. However, these systems capture gesture signals through accelerometers and gyroscopes [9,10,11], which require users to wear many sensor devices, leading to a suboptimal user experience. On the other hand, gesture recognition technology by vision sensors often relies on depth cameras to capture depth images of gestures [12,13,14] for recognition. Nevertheless, depth cameras are susceptible to ambient light interference, and depth images may contain substantial user information, posing risks of privacy breaches [15]. Additionally, these sensors may suffer from high power consumption and susceptibility to environmental factors [16].

The rapid development of mmWave radar sensors in recent years has provided new ideas for gesture recognition. MmWave radar is characterized by high frequency, high resolution, and low power consumption [17], which can provide accurate detections, making it possible to perform non-contact sensing and recognition of gestures, and it is relatively insensitive to common interfering factors such as occlusion, rain, and snow. Moreover, it can penetrate some non-transparent objects and identify gesture information behind occluded objects. Due to its high-frequency and high-resolution characteristics, it can capture the hand movement trend. Compared with camera-based gesture recognition technology [18], millimeter wave radar relies on electromagnetic waves to collect the characteristic information of the reflector, so it can prevent the disclosure of privacy caused by camera pictures and is not affected by light. Therefore, mmWave radar has apparent advantages in gesture recognition. For example, with millimeter-wave radar and a cane, it is possible to detect obstacles in complex environments, identify pedestrians and vehicles on the road, and assist blind people in traveling [19]. It is possible to monitor a worker’s vital signs, respiratory activity, and sitting position during work through radar technology and track his position, preserving individual privacy safely and cost effectively [20].

The most popular gesture recognition framework consists of two main parts: feature extraction and machine learning. The feature extraction could be via a range-Doppler map, micro-Doppler map and range-azimuth image. The most effective machine learning approaches are neural networks. The pioneer gesture recognition work by Google-designed mmWave radar (Soli) [21] utilizes the range-Doppler map as the training data. Lars et al. utilize the micro-Doppler map [22] to train the ResNet network combined with migration learning to correct the original network to complete the gesture recognition [23]. Shrestha et al. train the micro-Doppler features by LSTM and Bi-LSTM networks [24]. The networks were validated with an accuracy of more than 90%. The work by Alirezazad et al. [25] combines the range-Doppler map and the range-azimuth image to train a two-stream artificial neural network. An accuracy of 92.5% was achieved. The micro-Doppler map-based features are cluttered by complex environments, and they have impacts on model training and robustness.

In recent years, the Transformer model [26] has made some achievements in natural language processing (NLP) [27], computer vision (CV) [28], and other fields. The Transformer mode performs better gesture recognition than other neural networks for time series of radar data. The research in [29] achieves better results by introducing a Transformer for the sequence modeling of hand gestures. Biao Jin utilizes micro-Doppler maps to train the 2DCNN+Transformer model for gesture classification with 98% accuracy [30]. Kehelella et al. combined the Transformer with a convolutional encoder to propose a vision converter-based HGR architecture and achieved 98.3% accuracy [31]. Song combined convolutional and attentional mechanisms to collect gesture echoes using the MMWCAS radar data to generate hybrid feature–time maps of distance–time, Doppler–time, azimuth–time, and elevation–time, which were inputted into a DenseNet-CBAM network to recognize 12 micromanipulation gestures with 99.03% accuracy [32].

The current feature extraction mainly adopts a micro-Doppler map or range-Doppler map as the training data. However, this method suffers from the problems of massive data volume, too much redundant information, and insufficient feature extraction. By utilizing point cloud data as the input, we address several challenges compared to micro-Doppler images:

- Lower quality trajectory;
- Less gesture information;
- Highly sparse data;
- Irregular shapes of the point clouds at the same posture;
- Susceptibility to various noises and disturbances.

Despite these challenges, feeding the point cloud data into the TRANS-CNN network allows us to achieve an accuracy of 98.5%, which is equivalent to the level of gesture recognition accuracy achieved with micro-Doppler images as input. Furthermore, we can complete the gesture recognition within 1 s. The use of point cloud data effectively enhances the representation of gesture features, reduces the data volume per gesture (a dynamic gesture data in micro-Doppler images is around 2–3 MB, while our point cloud data have a size of less than 15 KB for a dynamic gesture), reduce data redundancy, and greatly improve gesture recognition speed, enabling real-time gesture recognition.

The innovativeness of our contribution is outlined below:

- In gesture recognition, point cloud [33,34] features instead of high-dimensional micro-Doppler features are used as the model’s input.
- The TRANS-CNN model combines multi-head self-attention mechanisms with a 1D convolutional network. Self-attention mechanisms extract global information from point cloud data, while the one-dimensional convolutional network extracts local information and the correlation and differences between sequences within the point cloud data. This effective differentiation allows for distinguishing between different types of gestures. The use of attention mechanisms combined with a one-dimensional convolutional network significantly reduces the overall complexity of the model, minimizes the utilization of hardware resources, improves the speed of gesture computation, and enables real-time gesture recognition.

The remainder of the paper is divided into six parts. The second part introduces the gesture recognition system, including point cloud detection and network structure design. The third part presents a feature extraction approach to build our training data. TRANS-CNN is described in the fourth part. The fifth part shows the experimental results, and the performance is evaluated by comparing different approaches. The last part concludes the paper.

## 2. Gesture Recognition System

**Figure 1:**
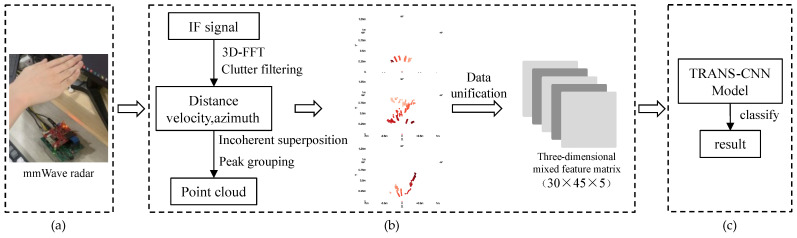
Framework of TRANS-CNN based gesture recognition for mmWave radar: (**a**) data collection; (**b**) radar echo signal processing; (**c**) gesture recognition.

**Figure 2:**
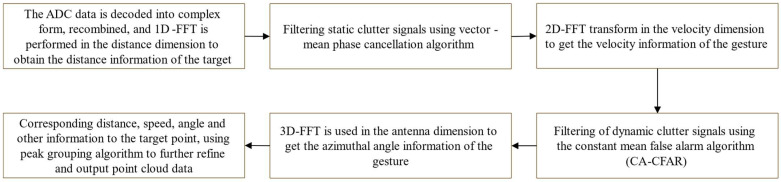
Point cloud acquisition process.

The framework of the gesture recognition system shown in Figure 1 consists of two main modules: feature extraction and TRANS-CNN model training. Our hand gesture data are captured by using mmWave radar. The detailed steps of gesture point cloud data acquisition are shown in Figure 2. The obtained point cloud data are used as the training dataset of the TRANS-CNN model. The design of our TRANS-CNN model is to dig up the mechanism between the time domain and spatial domain of the hand gesture data. Finally, the training process optimizes the mechanism and provides robust solutions for gesture recognition.

### 2.1. Feature Extraction of Point Cloud Data

**Figure 3:**
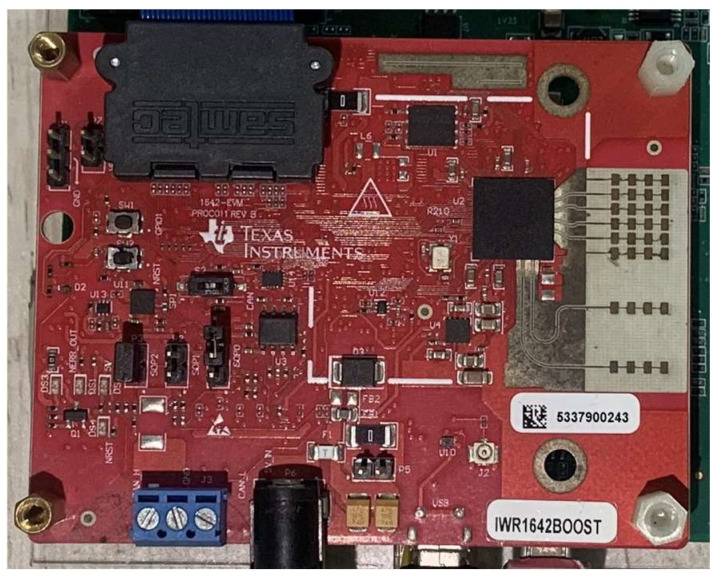
IWR1642 radar sensor.

As benchmarking, the TI Radar IWR1642 with 2Tx4Rx is used for data capture, as shown in Figure 3. The operating frequency range of the sensor is from 76 to 81 GHz, with a maximum bandwidth of up to 4 GHz.

The radar emits a Frequency-Modulated Continuous Wave (FMCW):

(1) $$s_{\tau }(t)=A_{\tau }\cos[\pi (2f_{0}t+St^{2})+\phi_{0}]$$

where $A_{\tau }$ is the amplitude of the transmitted signal, $f_{0}$ is the center frequency of the carrier, $S=B/T_{c}$ is the FMCW slope, B is the bandwidth, $T_{c}$ is the pulse width, and $\phi_{0}$ is the initial phase.

The echoed signal received after being subjected to delay and Doppler shift caused by gesture movement is

(2) $$s_{r}(t)=A_{r}\cos\pi [2f_{0}+S(t-t_{d})](t-t_{d})$$

(3) $$f_{r}=S(t-t_{d})+\Delta f_{d}$$

where $A_{r}$ is the amplitude of the received echo signal, $f_{r}$ is the frequency of the received signal, $t_{d}=2(R_{0}+vt)/c$ is the delay time of the echo signal, and $\Delta f_{d}=-2vf_{0}/c$ is the Doppler shift caused by the gesture movement.

The transmission signal and echoed signal are mixed through the radar’s built-in mixer, and then they undergo filtering by a low-frequency filter to remove irrelevant high-frequency components and noise, resulting in the extraction of a proper intermediate frequency (IF) signal:

(4) $$s_{IF}(t)=s_{\tau }(t)\times s_{r}(t)=\frac{1}{2}A\cos[2\pi (St_{d}-\Delta f_{d})+2\pi f_{0}t_{d}]$$

where $A=A_{r}\times A_{\tau }$ is the amplitude of the IF signal.

**Figure 4:**
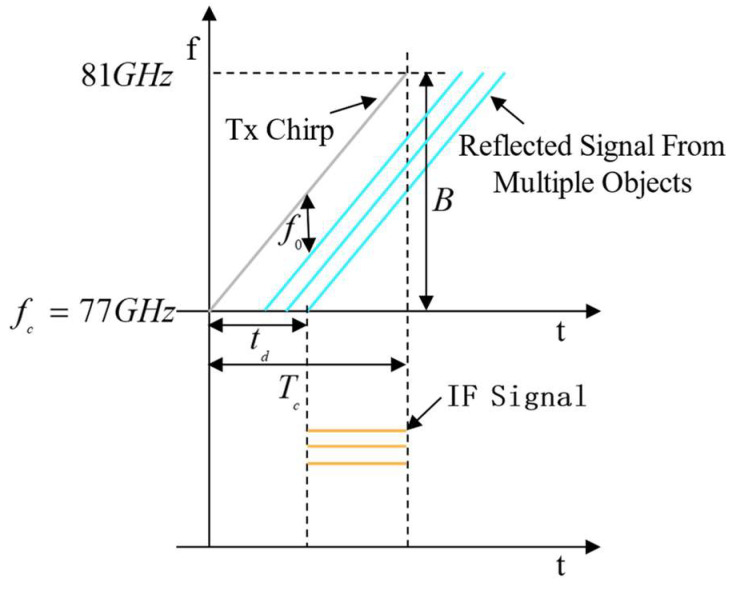
Relationship between transmitted signal, received signal, and IF signal. The radar transmits electromagnetic waves (Tx Chirp) at a specific frequency, and after a delay (caused by the gesture movement), it receives the signals reflected from multiple targets, which are mixed by the radar’s internal mixer to obtain an IF signal.

The relationship between the transmitted signal, received signal, and medium-frequency (IF) signal is shown in Figure 4.

Since the IF radar signal contains the motion information from hand and background objects [35], the background noise in the signal should be removed by the Vector Mean-Cancellation algorithm in Section 2.1.1, and the hand features should be detected by the CA-CFAR algorithm in Section 2.1.2.

#### 2.1.1. Clutter Removal

The captured IF Radar raw data are cluttered in a real-world environment. The environment includes stationary and moving objects.

- Static Clutter Filter

**Figure 5:**
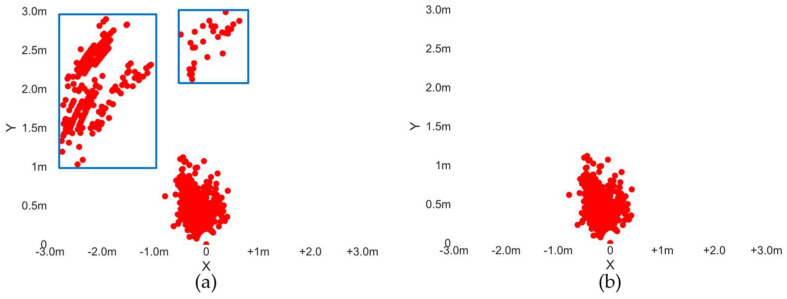
Comparison before and after static clutter filtering. (**a**) accumulated point clouds, where the red points inside the blue boxes correspond to the background targets behind hand; (**b**) red point cloud corresponds to the hand gesture. These static points inside the blue boxes in (**a**) are removed by the Vector Mean Cancellation algorithm,.

To address the echo signals caused by static clutter, this study applies a Vector Mean-Cancellation algorithm to the results of 1D-FFT to filter out static clutter in the echo signals. The core idea is to calculate the mean of the intensity for each Chirp and subtract this mean from the original Chirp intensity. This process yields data with static clutter removed. The point cloud after clutter removal is shown in Figure 5, where the blue region in the left image represents targets generated by static objects.

(5) $$R[rt,m,n]=D[rt,m,n]-\frac{1}{N}\sum_{i=1}^{N}D[rt,m,i]$$

where D[rt,m,n] is the data containing the clutter signal, m is the number of Chirps, and n is the number of sampling points for each Chirp.

- Dynamic Clutter Filter

Since the radar data are noised by the other body parts or pedestrians while gesture capturing, the static filtering process cannot completely filter out dynamic clutter. Therefore, the Constant-False-Alarm-Rate algorithm (CA-CFAR) [34] is used for range-Doppler radar images, which adaptively adjusts the decision threshold according to the dynamic clutter in the echo signal. CFAR detection uses the reference unit in the data to estimate the clutter noise and other parameters to obtain the threshold based on the estimated density. If a value exceeds the threshold in the detected data, it is considered that there is a target.

(6) $$H=\frac{K}{N}(\sum_{i=1}^{\left(N-M\right)/2}x_{i}+\sum_{j=\left(N+M\right)/2+1}^{N}x_{j})$$

where H is the unit’s threshold to be measured, K is the threshold for clutter estimation, N is the length of the distance dimension or velocity dimension, M is the length of the protection unit, and $x_{i}/x_{j}$ is the distance dimension or velocity dimension input data.

After the clutter removal, the classical 3D FFT is used for estimating the point cloud.

#### 2.1.2. Point Cloud Refinement

**Figure 6:**
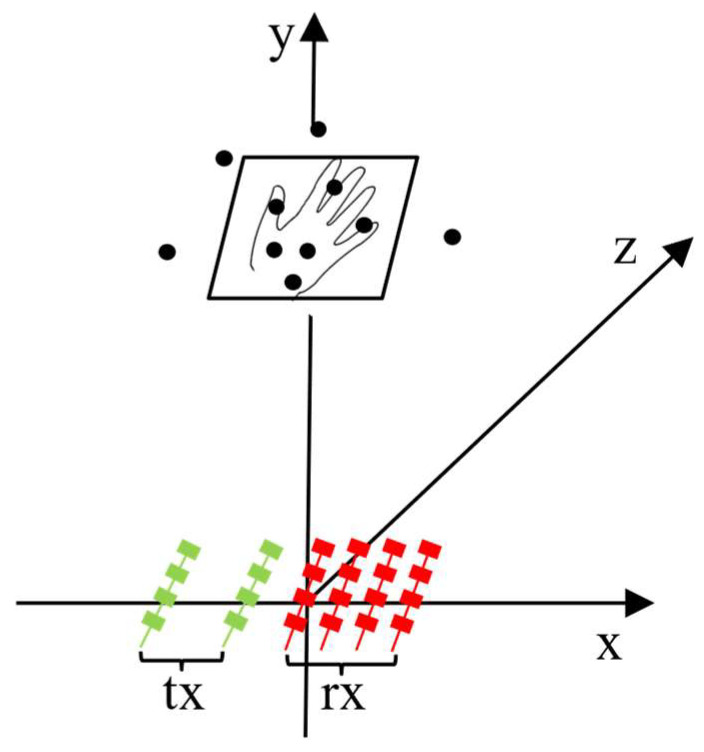
Hand gesture point cloud detection and peak grouping algorithm.

**Figure 7:**
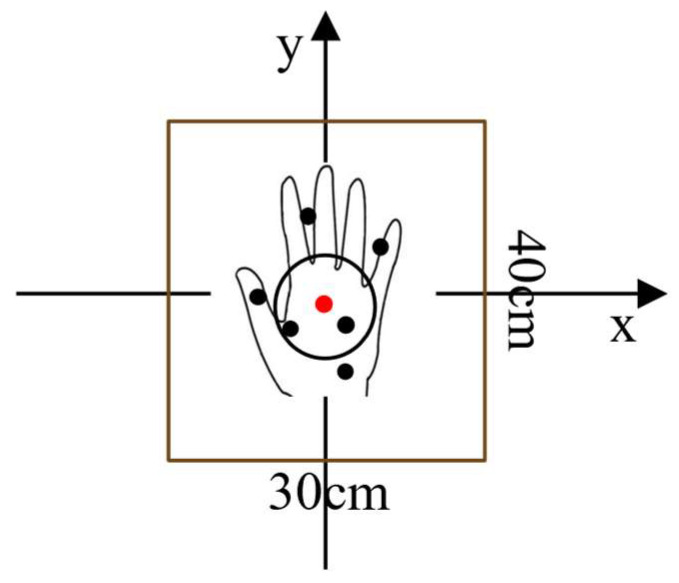
Collecting all the points in the defined hand area, and calculating the center position of the hand. The center position is marked as the red point.

In the actual processing of data, to fully demonstrate the effect of dynamic gestures, the false alarm rate of CFAR should not be set too small, which will therefore lead to some dynamic clutter still existing in the data after 2D CFAR, which is not conducive to the accurate recognition of gestures by the model. Therefore, the peak grouping algorithm [36] is used to group the internal target points of each gesture sample after CFAR. Target points that meet the requirements are retained, and those that do not are deleted. The specific process is outlined below:

(1) Locating the position of hand points by finding the target point with the max intensity.
(2) Searching for the three biggest points in the window of 40 cm × 30 cm, whose center is the position from step 1, as shown in Figure 6.
(3) Updating the center position by weighting these three points by step 2, as shown in Figure 7.
(4) Collecting all points that fall into the updated window centered at the new position by step 3.

**Figure 8:**
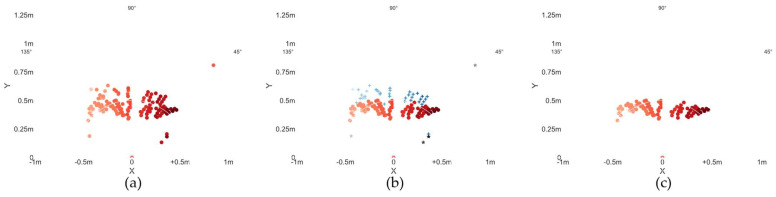
Clutter removal refinement by peak grouping for gesture “wipe from left to right”: The colors from light to dark represent the point cloud occurring time (from frame zero to the last frame). A plus sign indicates the invalid points. Pentagrams indicate points that are out of the moving area. (**a**) original cumulative point cloud; (**b**) peak grouping; (**c**) point clouds after peak grouping.

The effect of the gesture “hand wipe from left to right” to the right after grouping is shown in Figure 8.

### 2.2. TRANS-CNN Model Training

#### 2.2.1. Preprocessing of Training Data

When analyzing the collected point cloud data, it is found that the number of frames in each sample data and the number of target points in each frame are not the same, while the input of the model requires that the data format retains strictly consistent dimensions. The sample point cloud data are normalized in a matrix with 30 frames by 45 target points. Each point cloud datum has 5 features: x, y, range, Doppler, and azimuth. The frame cycle is 100 ms. If the sample data have a smaller matrix, the rest of the data matrix is padded with zeros; otherwise, sub-sampling is performed. Finally, the sample point cloud data represented as a 3D matrix with 30 frames by 45 points by 5 features are obtained.

#### 2.2.2. TRANS-CNN Model

**Figure 9:**
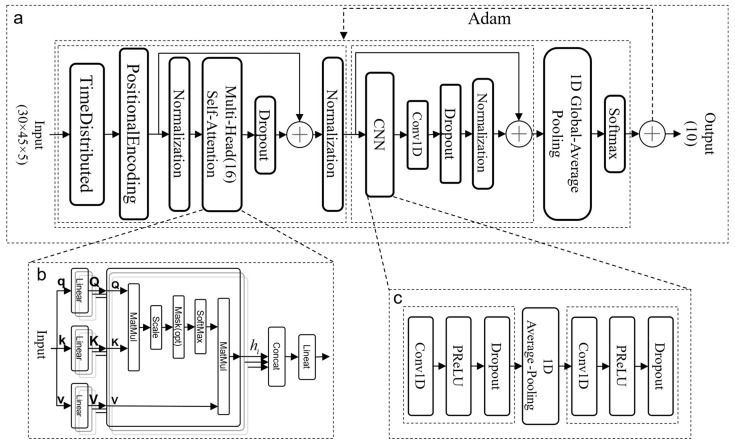
TRANS-CNN network structure: (**a**) overall network structure of gesture recognition; (**b**) multi-head self-attention mechanism based on scaled dot-product; and (**c**) serial 1D convolution module composed of two 1D convolution layers.

In 2017, the attention mechanism was first noticed and applied in Transformer [26]. Today, the attention mechanism has been widely used in NLP, CV, and other fields [27] and has achieved great success. However, for gesture recognition using point cloud data in this paper, the structure of the Transformer model is very complex and slow in computation. In contrast, this paper only employs the attention mechanism, reducing the complexity of the Transformer model by half, from the original O(n × d^2^) + O(m × d^2^) to O(n × d^2^), where n is the length of the input sequence, m is the length of the output sequence, and d is the dimensionality of the heads. Additionally, multi-head self-attention mechanisms are good at time sequential data and the lack of the spatial features in modeling. Adding one-dimensional convolutional networks strengthens the model’s attention with spatial features by using the point cloud data. The network structure of the TRANS-CNN model is shown in Figure 9. This part reviews the TRANS-CNN model.

- Attention Module

The structure of the attention module network in this paper is mainly improved based on the Transformer encoder network. It is divided into two pieces: temporal position encoding and the computation of dependencies between sequence targets.

The workflow of the time sequence position coding part is as follows: Firstly, the three-dimensional mixed feature tensor (30 × 45 × 5) is distributed and flattened in the time dimension through the Time Distributed function to ensure that all the data of each time frame participate in the calculation of the attention mechanism. Then, the Positional Encoding function is used to add nonlinear encoding values to the sequence using the standard position encoding method [26] which is used in our model, so that the model can better learn the relative relationship between different positions. Finally, it is copied and normalized by

(7) $$\tilde{X}=\frac{X_{i}-\bar{X}}{\sqrt{\sigma_{X}^{2}+K}}$$

where $\tilde{X}$ is the data after normalization, $X_{i}$ is the feature of the *i*th position of each frame, $\bar{X}$ is the mean of $X_{i}$ , $\sigma_{X}^{2}$ is the standard deviation of $X_{i}$ , and K is a constant.

Utilizing the attention mechanism based on scaled dot-product as shown in Figure 9b, continuous learning and computing the interdependence between different positions of target points in the target sequence are carried out. The variable A is duplicated and assigned to the query vector *Q*, the key vector *K*, and the value vector *V*, so as to realize the self-attention mechanism. The distinctiveness between different gestures is adequately extracted by

(8) $$h_{i}=\mathrm{soft}\max(\frac{Q\times K^{T}}{\sqrt{d_{k}}})\times V$$

where $h_{i}$ is the output of the *i*th attention header, and $d_{k}$ is the length of *K*.

Single-head attention has some limitations in extracting gesture features, and it is difficult to fully capture the gesture features in the sample data [37]. In contrast, in the multi-head attention mechanism, the *Q*, *K*, and *V* of each sub-network of the attention head are independent of each other, and different feature expressions can be learned in different subspaces. Therefore, this paper proposes a multi-head self-attention mechanism to concatenate the results of sub-networks of these single attention heads. Subsequently, a linear layer continuously learns the dependencies between different targets. The learned weight matrix is utilized to map the multi-head self-attention output to the input data’s dimensions. Including the multi-head attention mechanism in the model effectively improves the expressive power of the model.

(9) $$MH=MultiHead(Q,K,V)=Concat(h_{1},h_{2},\cdots ,h_{i})\;\times \;W$$

where W is the weight matrix learned by the linear layer, MH=MultiHead(Q,K,V) is the output of the attention module, and Concat(•) is the connection function.

- Serialization of 1D Convolution Modules

In the process of multi-head self-attention computation, the focus is on the dependencies between targets at different positions within a sequence, but less attention is paid to the dependencies between different sequences. Therefore, after the attention module, this paper introduces a one-dimensional convolutional network and constructs a serial one-dimensional convolutional module, the structure of which is shown in Figure 9c. It is used to capture the dependencies between different sequences.

A one-dimensional convolution operation is performed for each frame of data output from the attention module. The process utilizes Equation (14) to extract features between sequences in different time spaces using appropriate step sizes and convolution kernels. Subsequently, the results are input into an activation function for nonlinear transformation, and the extracted features are fed into an average pooling layer for down-sampling. Next, the down-sampled results are inputted into the next one-dimensional convolutional layer to perform the same feature extraction operation.

(10) $$\begin{array}{c} x_{i,j}^{n}=f(\sum_{\begin{array}{l} i\in 30\\ j\in 225 \end{array}}x_{i,j}^{n-1}\times w_{i,j}^{n}+b_{i,j}^{n})\\ y_{i,j}=Down(x_{i,j-1}) \end{array}$$

where f(•) is the activation function (PReLU), $x_{i,j}$ is the input data and the output data, i,j is the position of the target point, n is the first convolutional layer, $w^{n}$ is the weight matrix of the convolutional layer, b is the bias, Down(•) is the average pooling function, and $y_{i,j}$ is the pooled feature.

The output of the sequential one-dimensional convolutional module undergoes normalization through a one-dimensional convolutional layer and a regularization layer. Subsequently, a residual connection is employed to fuse the output with the input of the sequential one-dimensional convolutional module. Finally, the gesture is recognized by the global average pooling and a fully connected layer (Softmax).

## 3. Experimental Result

### 3.1. Radar Configuration

The IWR1642 mmWave radar from TI was used in this experiment, transmitting FMCW through time-division multiplexing mode (TDM-MIMO). The parameter configurations of the radar are detailed in Table 1. The distance resolution of the target was obtained from the calculations to be 0.039 m, and the radial velocity resolution was obtained to be 0.125 m/s.

The experimental platform also includes a computer with an I9-12900H processor, an RTX3060 (6G) graphics card, and 16GDDR5 RAM. The TRANS-CNN model was built using the TensorFlow-gpu 2.7.0 and Keras 2.7.0 frameworks.

### 3.2. Data Acquisition and Training Data Collection

**Figure 10:**
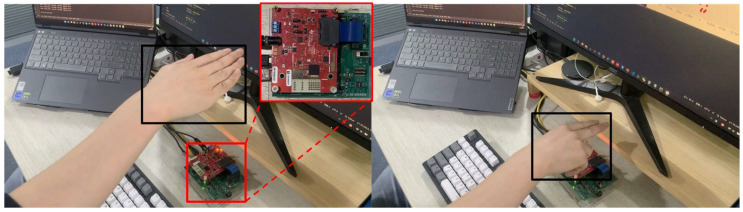
Two palm shapes used for hand gestures.

Before collecting the data, we investigated the gesture habits of 20 students in the lab. We counted the palm shapes they used when performing gestures, the range of motion of the gestures, and the trajectory of each gesture. We found that all 20 students used the two palm shapes as in Figure 8 to perform gestures, and the range of motion of their gestures were different, and the trajectories of the same gesture were not the precisely the same. Therefore, when we collected data, we stipulated that the radar was placed horizontally, and the gesture movement range was completed within 30~85 cm (±10 cm) in the radar boresight direction and −65~65 cm in the horizontal direction. At the same time, we collected data through different hand shapes with varying trajectories of motion of the same gesture. As an example of the wipe gesture, the gesture trajectory is not strictly in boresight and can be deviated by 5–10° to the left and right when collecting data. An example of the test setup and the palm shapes for gesture is shown in Figure 10.

**Figure 11:**
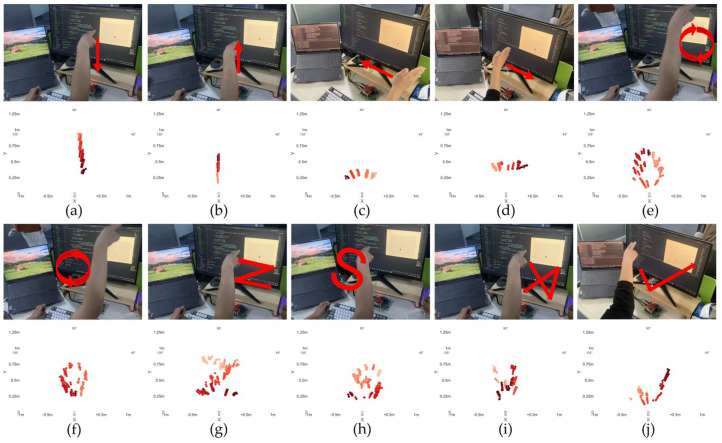
Gesture examples with corresponding cumulative point cloud: (**a**) wipe down (**down**), (**b**) wipe up, (**c**) wipe to the left, (**d**) wipe to the right, (**e**) rotating clockwise, (**f**) rotating counterclockwise, (**g**) drawing a Z, (**h**) drawing an S, (**i**) drawing an X, and (**j**) drawing a hook (√). For example, the gesture down indicates the hand moves from **top** to **bottom** facing the radar sensor. The points are the detections by the radar sensor.

To demonstrate the concept of our gesture recognition system, ten typical gesture movements are defined in our tests, including wipe up (up), wipe down (down), wipe to the left (left), wipe to the right (right), rotating clockwise (CW), rotating counterclockwise (CCW), drawing a hook (√), drawing an X, drawing a Z, and drawing an S. Five volunteers (3 men and 2 women) are invited to participate in the data collection. A total of 10,000 sets of sample data are collected for 5 participants, 10 gestures, 2 palm shapes, and 100 groups for each palm shape. Each dataset contains five significant features, resulting in a multi-dimensional point cloud dataset. A specific example of a gesture action and its corresponding multi-frame cumulative point cloud is shown in Figure 11. The colors from light to dark represent the movement direction of the gesture.

### 3.3. Recognition Results and Evaluation

The input data of the model are uniformly processed as 30 × 45 × 5, and the Epoch is set to 200. The Early Stopping function is used, and the model is developed to stop training and save the best model when the loss value of the model is no longer decreasing within 25 consecutive Epochs on the test set. The loss function uses the categorical cross-entropy function, which is widely used in multi-objective classification applications. The Adam optimizer is selected, and the learning rate was set to be dynamically adjustable. If the loss value is not decreasing within 10 Epochs on the test set, the learning rate is reduced to 20% of the current learning rate. The initial learning rate was set to 0.001. The data-splitting ratio is 70:30, which means 70% of the data is for training and 30% is for testing. All models in the experiment were trained using the above parameter settings.

**Figure 12:**
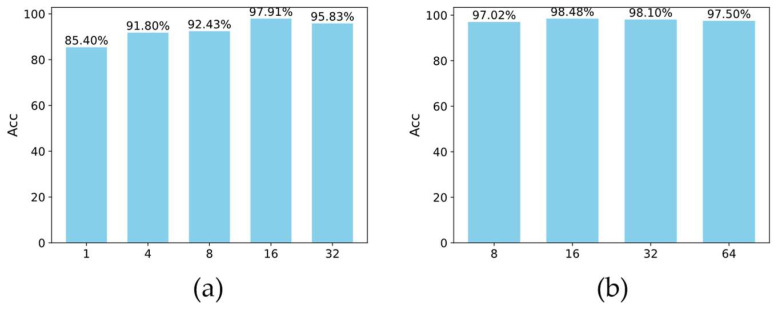
Model accuracy for different Num_heads and Batch_size: (**a**) represents the accuracy of different Num_heads corresponding to models, the horizontal coordinates of 1, 4, 8, 16, and 32 represent the number of heads (Num_heads) of the multi-head attention mechanism, and the vertical coordinates represent the accuracy of the model with different Num_heads; (**b**) represents the model’s accuracy corresponding to different Batch_size values, the horizontal coordinates of 8, 16, 32, and 64 denote the number of samples (Batch_size) at each training, and the vertical coordinates denote the accuracy of the model with different Batch_sizes.

To investigate the effect of multiple attention heads (Num_heads) on the model performance, 1, 4, 8, 16, and 32 attention heads were selected for the experiment, the default Batch_size was used to train the model, and the model accuracy was obtained to be 85.4%, 91.8%, 92.43%, 97.91%, and 95.83%, respectively, as shown in Figure 12a. It can be seen that the model accuracy achieves the maximum when the Num_heads is 16.

After setting Num_heads to 16, the effect of Batch_size on model performance is further explored; in the experiment, 8, 16, 32, and 64 are chosen to train the model, respectively, and the model accuracies are obtained as 97.02%, 98.48%, 98.1%, 97.50%, as shown in Figure 12b. Among them, the highest model accuracy is obtained when the Batch_size is 16.

**Figure 13:**
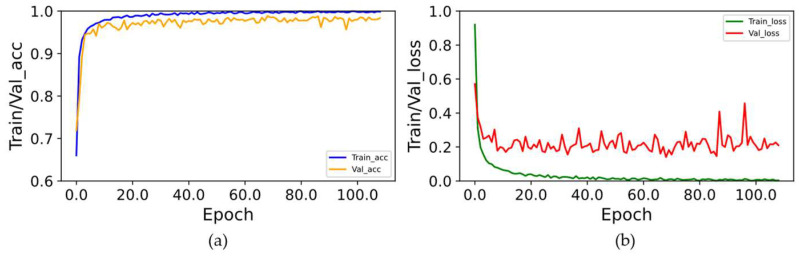
The Acc/Loss curve of the TRANS-CNN model: (**a**) model accuracy curve; (**b**) model error curve, with the horizontal coordinates denoting the Epochs on which the models were trained, the vertical coordinates of the (**a**) plot denoting the accuracy corresponding to each Epoch model, and the vertical coordinates of the (**b**) plot denoting the error values corresponding to each Epoch model.

While the Num_heads and Batch_size values are set to 16, the curves of accuracy and loss values are shown in Figure 13. During training, using the early stopping strategy, the model stops training at 109 Epochs, at which point we consider the model converged. It is clear from the curve that the model’s accuracy converges at about 98.5%. We test multiple n values, and the results show that the model’s accuracy is optimal when n is set to 25 (early stopping strategy: stop training when the loss value of the model on the test set is not decreasing within n consecutive Epochs; at this time, the model is considered to have reached convergence).

To evaluate the performance of the TRANS-CNN model, 1500 samples are collected again—150 samples for each gesture—and the confusion matrix is generated using the validation set simultaneously. The performance of the model is evaluated using precision, F1-score, recall, and the confusion matrix. Precision evaluates the model’s classification accuracy on the overall data, recall is used to assess the model’s ability to recognize positive samples, the F1-score integrates the relationship between precision and recall, and the confusion matrix visualizes the model’s classification effectiveness across different categories. The specific results are shown in Table 2. The average recognition accuracy of the ten gestures reaches 98.4%. The precision, F1-score, and recall of each gesture are statistically analyzed in Table 2. Prec (precision), Recall, F1-score (F1), and Acc (accuracy) are calculated as follows:

(11) $$Prec=\frac{TP}{TP+FP}$$

(12) $$Recall=\frac{TP}{TP+FN}$$

(13) $$F1=2\cdot \frac{Prec\cdot Recall}{Prec+Recall}$$

(14) $$Acc=\frac{TP+TN}{TP+TN+FP+FN}$$

where *TP* represents the number of instances where Class A is correctly identified as Class A, *TN* represents the number of instances where Class B is correctly identified as Class B, *FP* represents the number of instances where Class B is incorrectly identified as Class A, and *FN* represents the number of instances where Class A is incorrectly identified as Class B.

#### Validating Models with Public Datasets

**Figure 14:**
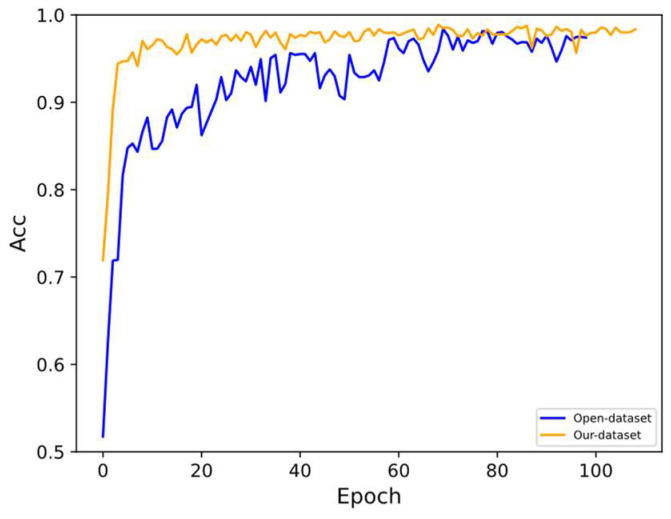
Model accuracy curves for different datasets on the test set with the horizontal coordinate denoting the Epoch of model training and the vertical coordinate characterizing the accuracy corresponding to each Epoch model.

The TRANS-CNN model is trained using the point cloud dataset disclosed by Piotr Grobelny and Adam Narbudowicz in IEEE [38], and the curves on the test set are plotted as shown in Figure 14. The TRANS-CNN model achieves an accuracy of about 98% in gesture recognition on this dataset, and the experimental results show that the TRANS-CNN model has good generalization ability.

### 3.4. Comparison of Different Gesture Recognition Approaches

#### 3.4.1. TRANS-CNN Compared with Other Models

Table 3 compares the TRANS-CNN model proposed in this paper with four models that use micro-Doppler images as input, including the Tesla model, Self-Attention, 2DCNN-Transformer, 8HBi-GRU, Dense-Net-CBAM, and VGG16. Ref. [18] uses optical flow to track the interest points in gesture videos and stores the tracked gesture motion as images for model input. Although the accuracy reaches 99%, the data processing is complicated, the data volume is large, and it cannot avoid the influence of occlusion and illumination changes. In contrast, this paper collects gesture information using radar, avoiding the impact of occlusion and illumination. Using point clouds as the model input simplifies data processing and reduces data volume. Compared to the Self-Attention model in Ref. [39], our TRANS-CNN model adds a one-dimensional convolutional network after the attention mechanism, fully considering the correlation and differences between sequences, resulting in a 3.7% increase in accuracy. Compared to Ref. [30]’s use of a 2D CNN network combined with a Transformer, our TRANS-CNN model reduces the complexity by half by removing the decoder and feedforward network parts of the Transformer and optimizing the 2D-CNN to a 1D-CNN network, while the gesture recognition accuracy increased by 0.5%. Compared to Ref. [40]’s 8HBi-GRU model, the TRANS-CNN model uses point clouds as input data. When the quality of the gesture trajectory is not as strong as the micro-Doppler image, the gesture recognition accuracy is improved by 0.3%, and gesture recognition can be completed within 1 s. Compared to the DenseNet-CBAM model designed in Ref. [32], which has an average accuracy of 99.03%, it does not fully utilize attention mechanisms to calculate the internal correlation of the sequence. Compared to the TRANS-CNN model in this paper, the robustness and generalization ability of the DenseNet-CBAM model is weaker, and the use of micro-Doppler images as input data involves more data processing, making it difficult to achieve real-time gesture recognition. Compared to the Tesla model in Ref. [34], the TRANS-CNN model combines multi-head self-attention mechanisms with one-dimensional convolutional networks to fully extract the global and local information of temporal sequence data and increase the attention between sequences, leading to a 1% increase in gesture recognition accuracy in an office environment.

In contrast, the TRANS-CNN model in this paper uses point cloud data as input. In comparison to micro-Doppler images, even with a lower quality trajectory and less gesture information carried, the recognition accuracy of the gesture can still reach 98.5% when the point cloud is sparse. Gesture recognition using point cloud data can be completed within 1 s, significantly improving the real-time performance of the gesture recognition system. Additionally, including attention mechanisms in the model enhances its robustness and generalization ability.

#### 3.4.2. Evaluation of Recognition on Simple Radar Sensor

**Figure 15:**
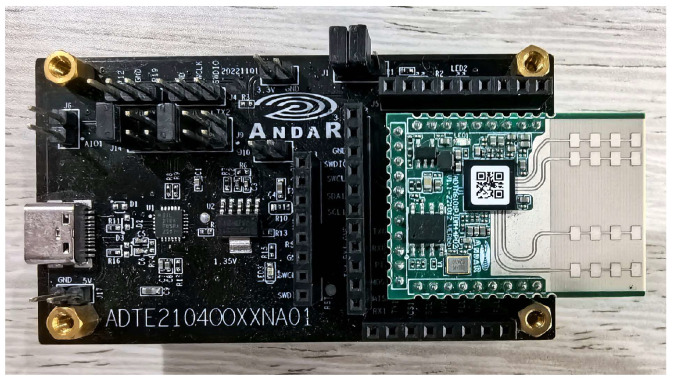
ADT6101 radar. The 2Tx2Rx is designed for our simple radar sensor to evaluate gesture recognition model.

Using the mmWave Radar ADT6101-4P with 2Tx2Rx developed in our lab shown in Figure 15, the data acquisition was carried out for the training and testing of the model. The radar parameter configuration is detailed in Table 4. The distance resolution of the target is calculated to be 0.1 m, and the radial velocity resolution is 0.28 m/s. In our test, the azimuth effective angle of the radar is about ±50°, and the elevation effective angle of the radar is about ±15° due to the four patches of the radar we developed in our lab. The hand motion range is from 30 to 85 cm (±10 cm) in boresight and from −65 to 65 cm in azimuth.

Due to the frame cycle of 100 ms, a fast hand motion is not allowed in our data capture. This limitation could be improved by increasing the frame cycle to 50 ms, and a real-time application is possible. For research purposes, a moderate hand motion is expected.

In this experiment, two volunteers were invited to perform data acquisition for four gestures, up, down, left, and right, with 120 groups for each gesture, totaling 480 groups of point cloud data. Subsequently, the dataset splitting ratio is 70:30 for TRANS-CNN model training and testing. The model converges quickly, and the specific test results are listed in Table 3. Using the ADT6101 radar for gesture recognition, it remains above 97%. The results indicate that the TRANS-CNN model can achieve significant performance on different radar devices.

**Figure 16:**
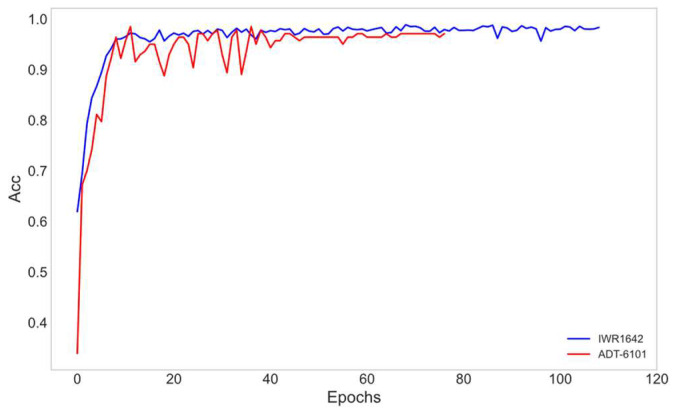
Acc change curves of IWR1642 (blue curve) and ADT6101 (red curve) with the horizontal coordinate denoting the Epoch of model training and the vertical coordinate characterizing the accuracy corresponding to each Epoch model.

To more comprehensively evaluate the model’s performance on different devices, the accuracy (Acc) variation curves of the IWR1642 and ADT6101 radar data are compared on the test set (see Figure 16 for details). The results show that the model exhibits high accuracy on both radars.

**Figure 17:**
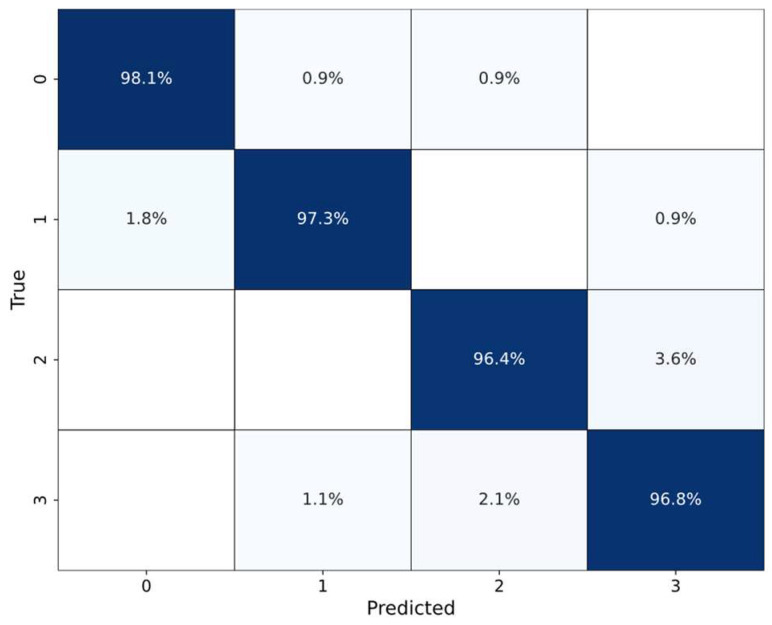
Confusion matrix of ADT6101 radar. The 0, 1, 2, 3, and 4 on the horizontal and vertical coordinates indicate up, down, left, and right, respectively. The higher accuracy is with darker color.

The confusion matrix for recognizing four gestures using the ADT6101 radar on the testing set is depicted in Figure 17. The confusion matrix illustrates that the recognition accuracy for both “up” and “down” gestures surpass 97%, while “left” and “right” gestures also approach 97%. Based on the actual gesture recognition performance, the TRANS-CNN model performs the gesture recognition task well in either radar.

## 4. Conclusions

In this paper, the TRANS-CNN model constructed by using point clouds as training data is proposed to solve the problems of high redundancy of information in micro-Doppler maps, insufficient feature extraction, and large amounts of data, which significantly improves the speed of gesture recognition and the possibility of model deployment. The gesture features are fully extracted using the multi-head self-attention mechanism and one-dimensional convolutional network to realize ten gestures. The robustness and generalization ability of the model is improved. Regarding to effectiveness, real-time, generalization ability, and gesture recognition accuracy, our proposed TRANS-CNN gesture recognition is better performed than other gesture recognition approaches. In the experiments, the proposed gesture recognition is evaluated on the benchmarking TI radar sensor, and it is also adapted to our lab-designed radar sensor, which is configured with the simple 2Tx2Rx antenna layout. The results show that our approach has potential applications in modern human–computer interaction. In our subsequent research, we consider implementing the TRANS-CNN model proposed in this paper in an embedded system. Another research direction is to reduce the frame cycle so that a faster hand gesture recognition is possible.

## Figures and Tables

**Table 1:** Radar parameters.

Radar Parameter	Value
Starting Frequency	77 GHz
Bandwidth	3.85 GHz
Frequency Modulation Slope	20 MHz/μs
Sampling Rate	2 MHz
Frame Cycle	100 ms
Sampling Points/Chirp	256
Chirp Number/Frame	32

**Table 2:** Confusion matrix of 10 gestures and their evaluation parameters.

	Predict	Up	Down	Left	Right	CW	CCW	Z	S	X	√	Recall (%)
True												
Up		149	1	0	0	0	0	0	0	0	0	99.3
Down		0	149	0	1	0	0	0	0	0	0	99.3
Left		0	0	149	1	0	0	0	0	0	0	99.3
Right		0	0	1	148	0	0	0	0	0	1	98.7
CW		0	2	2	0	146	0	0	1	0	0	97.3
CCW		0	0	0	0	0	148	0	2	0	0	98.7
Z		0	1	1	3	0	0	144	1	0	0	96.0
S		0	0	0	0	0	1	0	149	0	0	99.3
X		0	0	0	0	0	0	0	0	150	0	100
√		0	0	0	1	0	0	0	0	0	149	99.3
Prec (%)		100	97.4	97.4	96.1	100	99.3	100	97.4	100	99.3	Acc = 98.4
F1 (%)		99.7	98.3	98.3	97.4	98.6	99.0	98.0	98.3	100	99.3	

**Table 3:** Comparison of recognition accuracy of different models.

Model	Dataset	Radar	Number of Samples	Type of Gesture	Iteration Time/s	Acc/%
VGG16 [18]	Trajectory image	-	2000	10	-	99
Self-Attention [39]	micro-Doppler maps	BGT60TR24B	1600	8	-	94.8
2DCNN-Transformer [30]		IWR1642	2700	6	-	98
8HBi-GRU [40]		AWR1243	7200	12	2.41	98.2
DenseNet-CBAM [32]		MMWCAS	-	12	-	99.03
Tesla [34]	Point Cloud	IWR1443	12,097	5	-	97.5
TRANS-CNN		IWR1642	10,000	10	4.7	98.5
		ADT6101	480	4	1.3	97

**Table 4:** ADT6101 radar parameters.

Radar Parameter	Value
Starting Frequency	57 GHz
Bandwidth	1.5 GHz
Frequency Modulation Slope	41.7 MHz/μs
Sampling Rate	7.14 Mhz
Frame Cycle	100 ms
Sampling Points/Chirp	256
Chirp Number/Frame	64

## Data Availability

The datasets in this study will not be made public for legal/ethical reasons but may be made available upon reasonable request.
