# Correction: Zhang et al. TRANS-CNN-Based Gesture Recognition for mmWave Radar. *Sensors* 2024, *24*, 1800

**DOI:** 10.3390/s25133941

**Published:** 2025-06-25

**Authors:** Huafeng Zhang, Kang Liu, Yuanhui Zhang, Jihong Lin

**Affiliations:** College of Mechanical and Electrical Engineering, China Jiliang University, Hangzhou 310018, China; zhanghf@cjlu.edu.cn (H.Z.); zyh@cjlu.edu.cn (Y.Z.); jhlin@cjlu.edu.cn (J.L.)

**Text Correction**

There was an error in the original publication [1]. In Section 3.3, Paragraph 5, the sentences “Among them, the eight gestures of Up, Down, Left, CCW, draw S and draw √ have a precision of more than 97%. The accuracy rate for gesture recognition of Right, CW, and X reaches more than 96%, while the gesture recognition accuracy of Z is 94%.” were improperly expressed.

A correction has been made to Section 3.3, Paragraph 5:

To evaluate the performance of the TRANS-CNN model, 1500 samples are collected again—150 samples for each gesture—and the confusion matrix is generated using the validation set simultaneously. The performance of the model is evaluated using precision, F1-score, recall, and the confusion matrix. Precision evaluates the model’s classification accuracy on the overall data, recall is used to assess the model’s ability to recognize positive samples, the F1-score integrates the relationship between precision and recall, and the confusion matrix visualizes the model’s classification effectiveness across different categories. The specific results are shown in Table 2. The average recognition accuracy of the ten gestures reaches 98.4%. The precision, F1-score, and recall of each gesture are statistically analyzed in Table 2. Prec (precision), Recall, F1-score (F1), and Acc (accuracy) are calculated as follows:

(11) $$Prec=\frac{TP}{TP+FP}$$

(12) $$Recall=\frac{TP}{TP+FN}$$

(13) $$F1=2\cdot \frac{Prec\cdot Recall}{Prec+Recall}$$

(14) $$Acc=\frac{TP+TN}{TP+TN+FP+FN}$$

where *TP* represents the number of instances where Class A is correctly identified as Class A, *TN* represents the number of instances where Class B is correctly identified as Class B, *FP* represents the number of instances where Class B is incorrectly identified as Class A, and *FN* represents the number of instances where Class A is incorrectly identified as Class B.

**Error in Table**

In the original publication, there was a mistake in Table 2. The values of the three parameters “Prec, F1, and Recall” were incorrect. The corrected Table 2 appears below:

**Table 2:** Confusion matrix of 10 gestures and their evaluation parameters.

	Predict	Up	Down	Left	Right	CW	CCW	Z	S	X	√	Recall (%)
True												
Up		149	1	0	0	0	0	0	0	0	0	99.3
Down		0	149	0	1	0	0	0	0	0	0	99.3
Left		0	0	149	1	0	0	0	0	0	0	99.3
Right		0	0	1	148	0	0	0	0	0	1	98.7
CW		0	2	2	0	146	0	0	1	0	0	97.3
CCW		0	0	0	0	0	148	0	2	0	0	98.7
Z		0	1	1	3	0	0	144	1	0	0	96.0
S		0	0	0	0	0	1	0	149	0	0	99.3
X		0	0	0	0	0	0	0	0	150	0	100
√		0	0	0	1	0	0	0	0	0	149	99.3
Prec (%)		100	97.4	97.4	96.1	100	99.3	100	97.4	100	99.3	Acc = 98.4
F1 (%)		99.7	98.3	98.3	97.4	98.6	99.0	98.0	98.3	100	99.3	

The authors state that the scientific conclusions are unaffected. This correction was approved by the Academic Editor. The original publication has also been updated.
